# Venom On-a-Chip: A Fast and Efficient Method for Comparative Venomics

**DOI:** 10.3390/toxins9060179

**Published:** 2017-05-28

**Authors:** Giulia Zancolli, Libia Sanz, Juan J. Calvete, Wolfgang Wüster

**Affiliations:** 1 Molecular Ecology and Fisheries Genetics Lab, School of Biological Sciences, Bangor University, Bangor LL57 2UW, UK; 2 Venomics and Structural Proteomics Laboratory, Instituto de Biomedicina de Valencia, CSIC, Jaume Roig 11, Valencia 46010, Spain; libia.sanz@ibv.csic.es (L.S.); jcalvete@ibv.csic.es (J.J.C.)

**Keywords:** Bioanalyzer, *Crotalus*, on-chip electrophoresis, population-level variation, venom composition

## Abstract

Venom research has attracted an increasing interest in disparate fields, from drug development and pharmacology, to evolutionary biology and ecology, and rational antivenom production. Advances in “-omics” technologies have allowed the characterization of an increasing number of animal venoms, but the methodology currently available is suboptimal for large-scale comparisons of venom profiles. Here, we describe a fast, reproducible and semi-automated protocol for investigating snake venom variability, especially at the intraspecific level, using the Agilent Bioanalyzer on-chip technology. Our protocol generated a phenotype matrix which can be used for robust statistical analysis and correlations of venom variation with ecological correlates, or other extrinsic factors. We also demonstrate the ease and utility of combining on-chip technology with previously fractionated venoms for detection of specific individual toxin proteins. Our study describes a novel strategy for rapid venom discrimination and analysis of compositional variation at multiple taxonomic levels, allowing researchers to tackle evolutionary questions and unveiling the drivers of the incredible biodiversity of venoms.

## 1. Introduction

Animal venoms are highly complex cocktails of bioactive toxin peptides and proteins, tailored by natural selection to act on specific molecular targets in order to subdue prey or deter predators [1]. By virtue of their high specificity and potency, animal toxins have also been exploited as models for drug development and biodiscovery [2,3,4], structural biology [5,6], as well as in studies of molecular evolution [7,8,9]. Although the potential medical value of venom toxins has been contemplated since ancient times, large-scale in-depth characterization of venoms of diverse origins has only become feasible in the last decades. This is especially thanks to the unprecedented advances in “-omics” technologies, which have allowed the recognition of a new field of research, “venomics” [10]. This has led to the establishment of a standard approach for the characterization of venom proteomes [11,12]: briefly, crude venom is fractionated by reverse-phase HPLC (RP-HPLC), followed by SDS-PAGE of the RP-HPLC protein fractions, determination of molecular masses by mass spectrometry, in-gel tryptic digestion of protein bands, and internal amino acid sequence determination by nanospray-ionization CID-MS/MS of selected tryptic peptide ions. This bottom-up peptide-centric pipeline is the most widely used for qualitative and quantitative proteomic analyses of venom components from medically important snake species (see Table 1 in Calvete 2013) [13], but it has also been employed for comparative studies aimed at investigating patterns of venom composition between different species [14,15,16], as well as between distinct populations of the same species [17,18,19].

**Figure 1:**
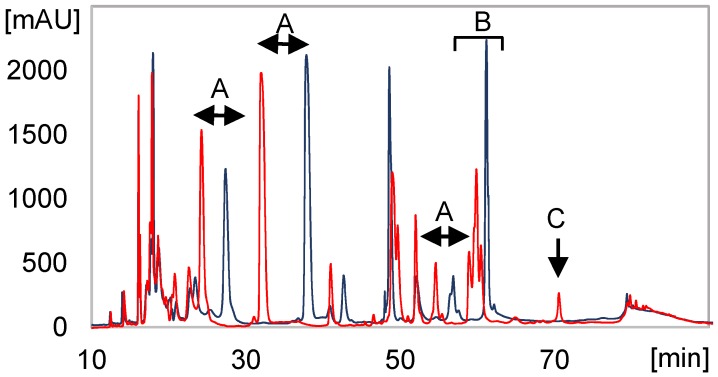
Overlapping of reverse-phase HPLC chromatograms of *Crotalus scutulatus* crude venom from the same individual sample, highlighting differences between independent runs. The same protein fractions can have different elution times (**A**); peak shapes (**B**); or intensities (**C**) between runs, resulting in difficult and unreliable scoring.

The extensive catalogue of snake venoms, in particular, has given rise to an interest in identifying the drivers and mechanisms behind this remarkable diversity and variation. Venom is an extremely complex phenotypic trait which has been shaped by natural selection over the course of evolution [1]. Gene duplication, positive selection, and protein neofunctionalization provide the evolutionary novelty that allows adaptation of venoms to different environments [20] or prey [9], as well as overcoming prey defenses against venom [21]. Because of these characteristics, venom represents an ideal model system for understanding ecological adaptation and prey coevolution [21,22,23,24,25]. However, investigating patterns of population-level variation requires large sample sizes to detect potentially subtle patterns and correlations with statistical confidence [21]. Thus, a method that allows rapid, detailed, consistent and repeatable characterization of multiple venom samples is needed. At present, RP-HPLC chromatograms are commonly used for this purpose. For instance, Saviola et al. [26] analyzed 34 venom samples from the Prairie rattlesnake, *Crotalus viridis*, from different geographic locations by comparing peak elution times and visual inspections of the chromatographic profiles. However, during a RP-HPLC run, proteins sometimes elute at different times, resulting in migration times that differ between runs of the same sample (Figure 1), and without the use of internal standards it is not possible to correct these discrepancies. Furthermore, proteins may elute differently (Figure 1), mainly because of fluctuations in room temperature. This is of particular importance in intraspecific comparisons, where variation between samples may be subtle. Finally, and most importantly, RP-HPLC run time is generally 90–120 min, limiting the throughput of massive and, at the same time, precise comparative analysis.

As an alternative approach, Gibbs & Chiucchi [17] analyzed population-level venom variation in the massasauga, *Sistrurus catenatus*, by means of comparisons of band presence/absence on Coomassie blue-stained SDS-PAGE. However, SDS-PAGE is a laborious method, and gel image analysis requires specific software, which is often not freely available, in order to score bands in an objective and standardized way.

It is clear that these methods, although essential for detailed characterization of venom components, present several limitations for the rapid identification of venom variation. Besides being long and laborious (technical details summarized in Table 1), these procedures are not able to compare large numbers of venom profiles in a fast, reliable, and consistent way.

**Figure 2:**
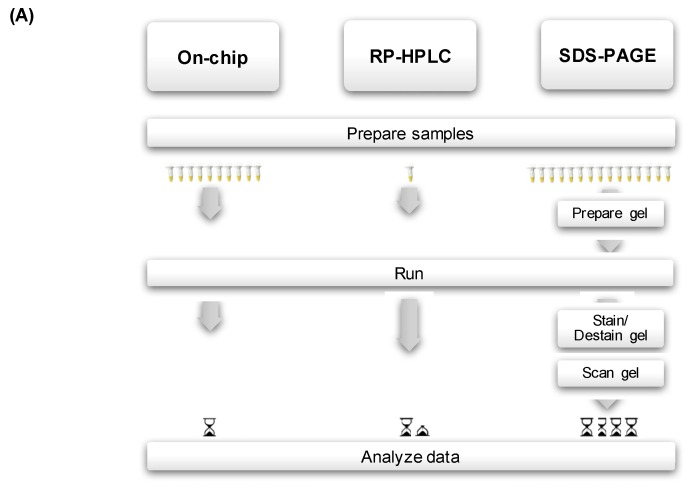

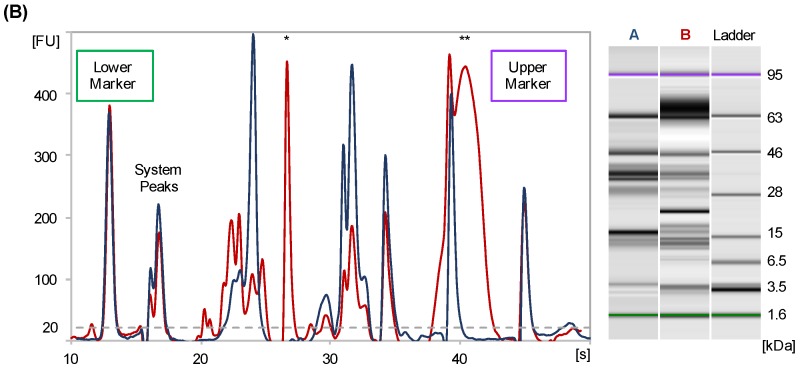
(**A**) Flowchart summarizing the steps involved in protein analysis of crude venom samples by means of on-chip electrophoresis, RP-HPLC and SDS-PAGE; (**B**) Example of two overlapped electropherograms (**left**) and the corresponding digital gel (**right**) from on-chip electrophoresis on the Agilent Bioanalyzer. Each sample is loaded with two internal standards: a “lower” (1.6 kDa) and “upper” (95 kDa) markers are used to align the electropherograms. The migration time of each peak is used to create the marker “bin” (in grey). All peaks falling in the bin are scored as present (1). Here we used venoms from *Crotalus scutulatus* type A (blue) and B (red) (refer to ‘Intraspecific variation’ for details); the latter has two distinctive peaks at approximately 23 kDa (*) and 67 kDa (**) likely corresponding to PI-SVMPs and PIII-SVMPs.

A new approach with great potential for the analysis of large numbers of crude venom samples in a fast, easy and reproducible way is by using the Agilent 2100 Bioanalyzer. This ‘lab-on-a-chip’ technology employs microfluidic capillary gel electrophoresis with laser-induced fluorescence detection for protein separation and quantitation within a disposable glass chip, where fluorescence intensities of proteins are measured as a function of their migration times. Venom samples are loaded into wells etched on the glass chip, and through a network of microchannels they move to the separation channel where individual components are electrophoretically separated. Proteins are detected by their fluorescence and translated into gel-like images (as bands), and electropherograms (as peaks) (Figure 2). Besides providing a fast, ready-to-use and easy-to-handle procedure, and minimizing reagent and sample usage, the Bioanalyzer corrects for varying injection efficiencies for individual samples by including internal standards (“lower” and “upper” markers) which are used to align each sample with the standard ladder. An extremely useful feature for profile comparisons is the possibility of overlay the electropherograms of multiple individual samples, thereby yielding precise alignments and migration times (Figure 2).

Here, we test the potential of this technology for large-scale comparative venomics using a panel of snake venom samples. Specifically, we tested the ability of the Bioanalyzer to: (1) efficiently separate crude venoms of distinct snake species; (2) detect large-scale variation in venom profiles at the population level; and (3) separate RP-HPLC fractions as an alternative to SDS-PAGE polyacrylamide gels to detect individual toxin proteins. Our results led us to report a robust and automated pipeline to generate reliable binary matrices of venom profiles which can then be used for a wide array of subsequent analyses.

## 2. Results and Discussion

### 2.1. Venom On-a-Chip

Our results demonstrate the ability of the ‘lab-on-a-chip’ technology coupled with the Agilent Protein kit to successfully separate complex protein mixes such as animal venoms. Here, we used the Protein 80 kit to analyze snake venoms in the range of 5–80 kDa; however, other size ranges (e.g., 14–250 kDa) are available, allowing the analysis of venoms from other animal species.

Across all snake species analyzed in this study, the average number of peaks per individual venom was 12, and overall standard deviation of an individual peak migration time was minimal (mean across all markers = ±0.14 s). To ensure reliability of this technique, we compared duplicate runs and counted the number of scoring mismatches for each marker. After removing the markers with two or more mismatches, the overall error rate decreased from 2.68% to 1.15%. Typically, genotyping studies based on binary markers such as AFLP have error rates of 2–5% [27], thus we can confidently conclude that our scoring was reproducible and reliable.

Although we observed peaks beyond the upper marker at 95 kDa, we did not score peaks above this threshold, as they might not align perfectly with the markers and thus would be less reliable and prone to error. One solution would be to use the Protein kit 250 kDa to capture toxins of molecular weight higher that 95 kDa. However, for our present analysis, the 5–95 kDa range is enough to capture the most highly-expressed and variable toxin proteins such as PLA_2_, SVMPs, CTLs, and myotoxin [28].

### 2.2. Interspecific Variation

We tested whether our method was able to efficiently discriminate venom profiles by analyzing the venoms of ten snake species including pit vipers of the genus *Crotalus* (*C. atrox*, *C. pyrrhus*, *C. scutulatus* and *C. viridis*), true vipers of the genus *Echis* (*E. borkini*, *E. carinatus sochureki*, and *E. coloratus*) and the African puff adder (*Bitis arietans*), and an elapid, the monocled cobra (*Naja kaouthia*). The final binary matrix was composed of 52 polymorphic markers. *C. scutulatus* type A and *E. coloratus* had the lowest number of peaks per individual profile (both 7 peaks), whereas *C. scutulatus* type B and two other *Crotalus* species, namely *C. viridis* and *C. pyrrhus*, had the highest number of peaks per profile (18, 16 and 15 respectively).

**Figure 3:**
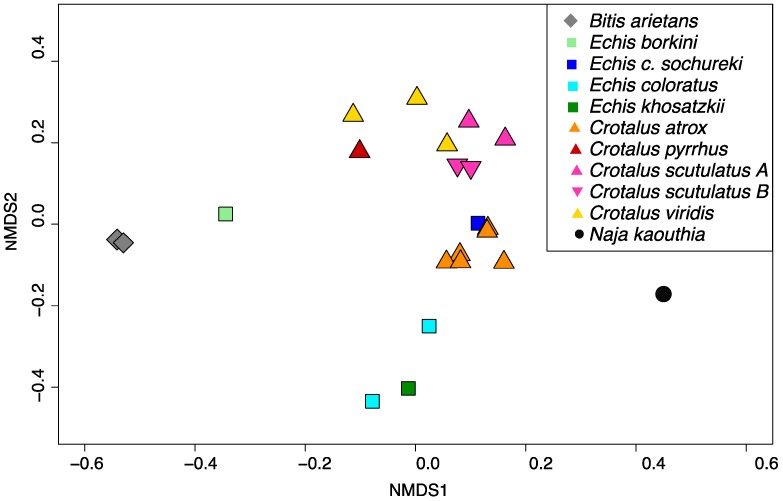
Non-metric multidimensional scaling (NMDS) of venom profiles across multiple snake species. Overall Stress value for the best-fit three-dimensional representation of the data was 0.08, thus the plot represents well the differences between venom profiles. Ordination stress value = 0.08.

As Figure 3 shows, this method successfully discriminated venoms from different species and revealed some interesting patterns. All rattlesnake species were close together in the ordination space, suggesting an overall common pattern in this group, despite the differences in venom activity between type A and type B venoms. Many venom components are indeed common to most rattlesnake species, and a high degree of sequence homology exists among specific components [28]. In contrast, *Echis* venoms were more scattered and they were all well separated from the rattlesnake venoms, except for *E. c. sochureki* which overlapped with *C. atrox*. It is well known that *Echis* vipers show extreme inter- and intraspecific variation [24,29,30] which is also reflected in failure of cross-neutralization of antivenoms [30,31,32], and may reflect diet-specific venom activities [24]. These differences are reflected in the wide scatter of venom profiles in the NMDS analysis.

We thus demonstrated that the on-chip technology can be used to rapidly and easily screen for overall variation and general patterns of venoms of different snake species.

### 2.3. Intraspecific Variation

**Figure 4:**
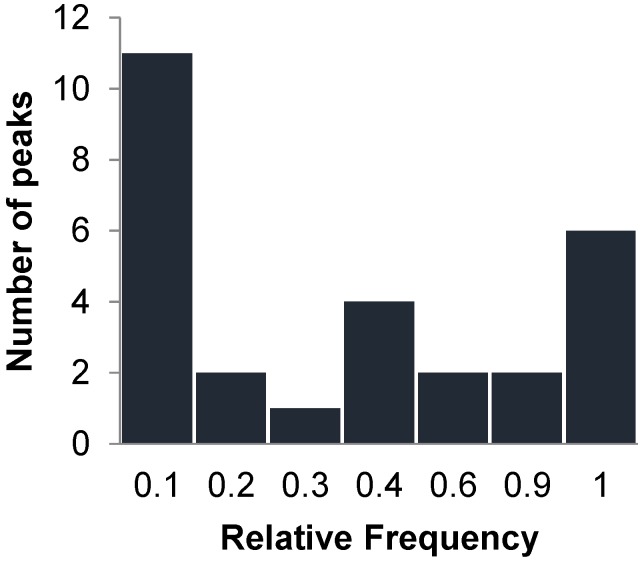
Frequency distribution of protein peaks across all venom samples of *Crotalus scutulatus*.

We further analyzed the venoms of 98 *C. scutulatus* rattlesnakes, to test the ability of the on-chip method to detect patterns of intraspecific venom variation. Initially, we used 28 duplicate samples to calculate standard deviations for each distinct peak, which then define the corresponding bin sizes. We then scored all the remaining samples by using our size-based criteria and recorded a total of 40 markers; of these, 12 were excluded as either difficult to score or prone to error (see above). The average number of peaks per individual venom was 12, with the least diverse profiles having seven peaks and the most diverse with 18. Peak frequencies were variable across all samples with the majority at low frequencies (Figure 4). Four markers were fixed (i.e., present in all the venoms); thus, they were removed for subsequent intraspecific analysis as uninformative, whereas the remaining 24 polymorphic peaks were kept. The observed bimodal distribution suggests that most venom proteins fall into two categories: those that are consistently expressed in the venom regardless of geographic location, and those that tend to be less frequently expressed, likely from snakes of a specific area or population. A similar pattern was also observed in a population-level analysis of *Sistrurus c. catenatus* venom variation [17].

**Figure 5:**
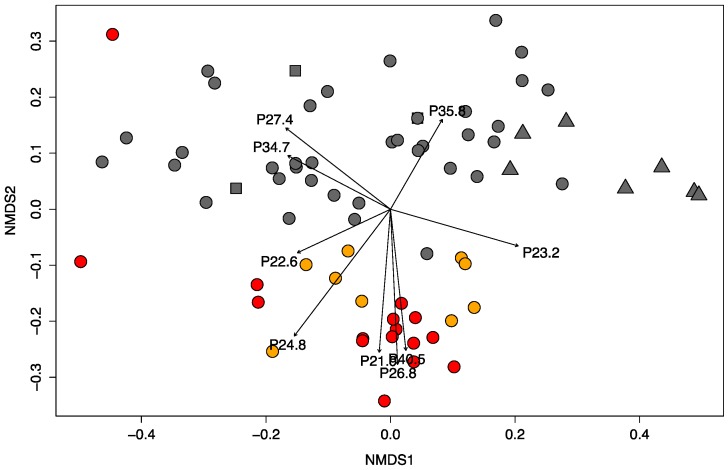
Ordination (NMDS) plot of *Crotalus scutulatus* venoms from California (square), Arizona (circle), and Texas (triangle). Venoms identified as type A are in dark grey, type B in red, and intermediate A + B in orange. Venom peaks are fitted onto the ordination (only markers with *p*-value < 0.001 are represented here). The arrows show the direction of the increasing peak frequencies, and the length is proportional to the correlation, i.e., strong correlations have longer arrows. Ordination stress value = 0.11.

In order to test if the venom variation captured by this method reflected the variation known to occur in this species, not only in terms of venom-type dichotomy (type A or B), but also in terms of subtle and more comprehensive variation [18], we first identified all venom phenotypes (i.e., unique venom profiles), and then we calculated pairwise similarities for the ordination analysis. Across 98 venom profiles, we identified a total of 71 unique phenotypes, 57 of which were singletons (i.e., unique profiles), revealing an extreme variation at the individual level. Three markers seemed to be highly correlated with venom type B as they showed consistent patterns of relative abundance with all phenotypes characterized by the presence of SVMPs as detected by fractionation of those individual venoms by RP-HPLC. In particular, we believe that peak P40.5, which includes proteins of approximately 67 kDa, represents PIII-SVMPs (which are generally ~43–60 kDa), and peak P26.8 (~23 kDa) represents PI-SVMPs (Figure 2). The correlation between these markers and venom type B was also observed in the NMDS analysis when we analyzed which markers contributed the most to the ordination plot. Peaks P40.5, P26.8 and P21.9 were indeed highly significantly correlated with the ordination space (*r*^2^ = 0.62, 0.75 and 0.64 respectively, *p*-value = 0.001), and their vectors pointed in the direction of venom type B individuals (Figure 5).

NMDS analysis also showed a much greater variation in the venom profiles that goes beyond the simple dichotomy, although a clustering of venoms type B can be observed (Figure 5). Interestingly, venoms type A showed a much wider variation compared to venom B, which, overall, clustered quite closely together, suggesting that, even though the Mojave toxin (MTX) is the major toxin characterizing these venoms, there is an array of other proteins that are selectively expressed across the geographic distribution investigated, as has been previously suggested [18].

We conclude that the Bioanalyzer can be a fast and reliable method to detect and investigate venom variation. The binary matrix obtained following our pipeline can be used to measure various diversity estimates, e.g., Shannon diversity index, and pairwise dissimilarities, which are not only informative regarding the overall variation and diversity of intraspecific venom composition, but can also be used as response variables for testing correlations with, for instance, population structure, environmental variables, habitat complexity or diet spectrum. It thus offers a great potential for rigorous statistical testing of multiple correlates.

### 2.4. Bioanalyzer with RP-HPLC Fractions

**Figure 6:**
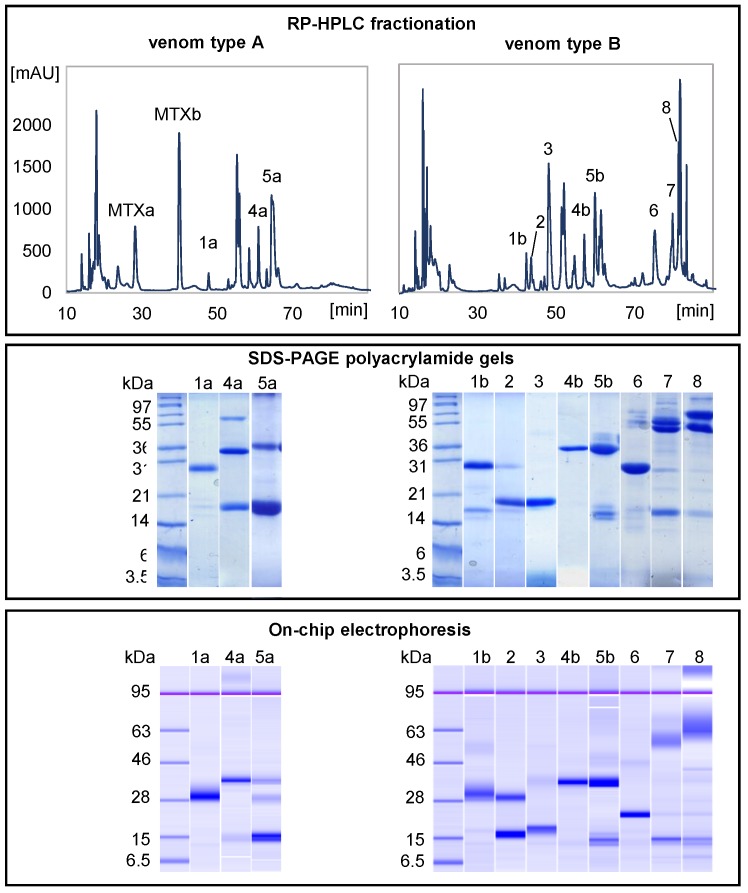
Comparison of reverse-phase HPLC venom fractions (**top panel**) separated by traditional SDS-PAGE (**middle**) and the on-chip Bioanalyzer (**bottom**) using *C. scutulatus* venom type A (**left**) and B (**right**). Only those fractions with the most relevant differences between the two venoms are shown. Molecular mass markers are at the left side of each gels. Proteomic-guided assignment of the chromatographic fractions to toxin families and relative molecular weights are shown in Table 2.

Next, we tested the possibility of using the Bioanalyzer in conjunction with previously fractionated venom samples to detect the presence or absence of specific toxins in the venom. Complex mixtures of proteins, such as venoms, generally require multiple fractionation steps in order to isolate individual proteins. Hence, chromatographic fractions of crude venoms are generally run on polyacrylamide gels to separate the toxins by their apparent molecular weights [11,12]. Although SDS-PAGE is mandatory to recover the individual bands for downstream MS/MS sequence analysis, it may not be ideal for detecting whether the product of a specific gene or isoform is secreted into the venom. In some rare cases, one HPLC fraction may harbor only a single proteoform, and thus the presence or absence of this toxin species can be easily discerned from the venom chromatogram, for example the MTX peaks (see Figure 6). However, in most cases, multiple proteins of disparate molecular weights co-elute in the same fraction.

We thus collected RP-HPLC fractions from venoms type A and B of the Mohave rattlesnake, ran them both on polyacrylamide gels and on the Bioanalyzer chip, and identified the protein family by CID-MS/MS. We focused on those protein fractions showing variation and marked differences between the two venom types as the most relevant for the analysis. Then, we compared the molecular weights estimated by the Bioanalyzer with those from the polyacrylamide gel, with the derived molecular weights from their amino acid sequences and, for a limited number of fractions, with mass spectrometry data. The migration behavior of venom proteins on the Bioanalyzer was comparable to that on SDS-polyacrylamide gels and, in some instances, the on-chip electrophoresis was able to discriminate proteins of similar masses as two distinct bands instead of just one, as in the SDS-PAGE gel (Figure 6). However, some venom proteins migrated at higher molecular weights than expected (Table 2); an exceptional case was fraction 4a, where the upper band in the SDS gel is around 66 kDa, whereas in the Bioanalyzer it is above the 95 kDa marker, with an estimated size of 109 kDa (Figure 6, Table 2). Such migration behavior on chip electrophoresis is known for proteins that have the tendency to form aggregates, such as caseins in milk [33], or as a result of other chemical properties, such as glycosylation, phosphorylation pattern and overall hydrophobicity, which can influence protein structure and interaction with the gel matrix during separation [34,35]. For instance, this effect is very prominent for k-casein, which shifts from an expected 19 kDa to approximately 46 kDa [33]. Some of the venom proteins seem to show a similar behavior; it is known indeed that toxins form poly-dispersed aggregates in the venom [36], for instance the Mojave toxin basic chain (see RP-HPLC fraction 29 in Figure 3 of Massey et al.) [18]. Post-translational modifications, including glycosylation, phosphorylation, disulfide bond formation and proteolysis, are known to alter the conformation and function of snake venom proteins, and to contribute to the overall diversity and variation of venom composition [30,36,37]. Such mechanisms could thus also explain the observed shift in apparent molecular weight. Glycosylation can in fact affect on-chip migration; this has been shown for glycoproteins with varying degrees of glycosylation, as N-glycans interfered with detergent attachment, resulting in flawed values of seemingly higher molecular weights, thus affecting on-chip migration [34,35]. Nevertheless, the shift in apparent molecular weight is a known effect when using on-chip protein electrophoresis and it is reproducible, and therefore actually helps with resolution and subsequent detection and quantitation of the individual proteins.

## 3. Conclusions

Our study has demonstrated that on-chip electrophoresis offers a fast, standardized, reproducible method for rapid analysis of compositional variation of crude animal venoms between different species, but especially for detecting subtle intraspecific variation at the population level. The main advantages over the traditional polyacrylamide gel electrophoresis is the elimination of time-consuming procedures and the ease of data processing and analysis: load samples, run analysis and view data. It produces a high quality dataset which can be used for multivariate analysis and testing of a set of ecological and evolutionary questions regarding the drivers of the observed variation in venom composition. Great advances in proteomics techniques have allowed for extensive characterization of venoms from a large number of snake species and it has revealed an incredible variation at all taxonomic levels, from between species to intraspecific differences within and between populations, as well as individual ontogenetic differences (reviewed in [13]). However, while much information on the biochemical and molecular composition of these venoms is becoming available, we still know very little about the drivers of this variation and the ecological correlates influencing the composition of venoms [38]. The method described herein thus offers a fast and reproducible approach for performing analyses of large numbers of samples on a scale that has rarely been attempted before, thus enabling the generation of large datasets and a strong statistical approach to their analysis. The on-chip electrophoresis can also be combined with RP-HPLC fractionation for a more detailed analysis of presence/absence of target toxins, and thus to relate phenotypes with genotypes and understand better the intrinsic factors governing venom variation. Finally, it has great potential as a complementary method to cDNA libraries or next-generation sequencing for discovery of new venom components, and it can be used for monitoring the stages of the toxin purification process, and for quality control and testing of antivenomics profiling [39].

## 4. Materials and Methods

### 4.1. ‘Lab-On-a-Chip’ Setup

First, in order to find the optimal starting conditions, we tested different amounts (1, 2, 4, 8, 16 and 20 μg) of venom from the Mohave rattlesnake, *Crotalus scutulatus*. As 16 μg yielded the best results—i.e., sharp and sufficiently, but not excessively, high peaks—we then used this for subsequent samples. Dried venoms were re-suspended in ddH_2_O to a concentration of 4 μg/μL and centrifuged for 10 min at 16,300× *g*. Samples were reduced with β-mercaptoethanol and prepared using the Agilent Protein 80 assay kit (Santa Clara, CA, USA) following manufacturer’s instructions. Most snake venom proteins have molecular masses in the range of 3–60 kDa; hence, we used the Protein 80 kit which is suggested for separation of proteins in the 5–80 kDa range. Data evaluation was performed using the freely available Agilent 2100 Expert software (version B.02.08.SI648, Santa Clara, CA, USA). All samples and the ladder were checked, and both internal markers adjusted if incorrectly assigned so that by overlapping all samples, the internal markers would all be perfectly aligned (see Figure 2).

### 4.2. Interspecific Variation

To test whether our method can efficiently discriminate different venoms, we first analyzed crude venoms from ten snake species, namely: two individuals of *Bitis arietans* from South Africa, two *Echis coloratus* from Oman and Saudi Arabia respectively, one *Echis carinatus sochureki* from Turkmenistan, one *Echis borkini* from Saudi Arabia, one *Echis khosatzkii* from Oman, six *Crotalus atrox*, one *Crotalus pyrrhus* and four *Crotalus scutulatus*, all from Arizona, three *Crotalus viridis* from New Mexico, and one *Naja kaouthia* from Thailand. We ran each venom sample in duplicate and scored all the peaks above 20 fluorescent units (FU). For each species separately, the migration times of each distinct peak in individual electropherograms were used to calculate the migration range, or “bin” size, for each marker (see Figure 2 for an example). Species-specific bin sets were then overlapped and merged to create one unique dataset for all species. Each electropherogram was scored to generate a presence (1)—absence (0) matrix which was used for subsequent analysis (see below).

### 4.3. Intraspecific Variation

For the population-level analysis, we used the venom of 98 individuals of *C. scutulatus* from California, Arizona and Texas. In the south-western USA, this snake species shows extreme geographic variation in venom composition [18,40,41]: venoms from most of the range are characterized by a neurotoxic heterodimeric phospholipase A_2_ (PLA_2_), “Mojave toxin” (MTX), and are rich in serine proteinases (SVSPs) and other PLA_2_ molecules (venom type A); however, in central Arizona, venoms are characterized by the absence of Mojave toxin but are rich in metalloproteinases (SVMPs), disintegrins, C-type lectins (CTLs), SVSPs and PLA_2_ (venom type B). Other phenotypes exist, such as intergrade venom types (A + B), and multiple combinations of either type A or B with other important toxins [18], for instance a myotoxin homologous to crotamine from the South American rattlesnake, *Crotalus durissus*. The Mohave rattlesnake thus offers an ideal model for testing our method as it offers a known strong pattern of geographic variation in venom composition.

First, we ran a subset of 28 samples multiple times (2–3 replicates). Similarly to the interspecific analysis, we used the migration times of each peak in the duplicates to create the bins. All individual bin sets were then overlapped and merged to create one unique bin set for the subset samples. Subsequently, we ran all the remaining samples and scored the peaks using the previously originated bin set. When a peak fell outside of any existing bin, we would re-run the sample to check whether the peak was shifted simply due to migration efficiency. If the peak was still outside of any available bin, a new additional bin was created. This procedure was used to generate a binary matrix for all markers. To test whether our procedure was able to discriminate between different venom phenotypes and capture overall venom variation in this species, we ran the venom samples through RP-HPLC to test for the presence of MTX and SVMPs (whose peaks are well characterized in rattlesnake chromatograms) [18]. Approximately 0.7 mg of venoms were separated on an Agilent 1100 system (Santa Clara, CA, USA) using a Teknokroma Europa 300 C18 column (250 × 4 mm, 5 μm particle size, Teknokroma, Barcelona, Spain) eluting at 1 mL/min with a linear gradient of 0.1% TFA in water and acetonitrile. Representative type A and B venom samples were selected for further proteomic analysis (see below).

### 4.4. Error Rate

Several precautions were taken to ensure reliability of the protocol. First, we used the subset of duplicated samples to exclude unreliable markers (peaks that were unstable or difficult to score) from the dataset. Each peak scored as present in one electropherogram and absent in the other one of the same individual was counted as a mismatch. Markers accumulating ≥ 3 mismatches were considered as prone to scoring errors and were discarded. Additionally, we used the number of mismatches to calculate the mismatch error rate. Using the same principle of studies based on molecular markers such as AFLP or microsatellites [42], we calculated the error rate as the ratio between observed number of mismatches and total number of comparisons. Reporting error rates is a key measure to allow evaluation of data quality and assessment of the reliability of the published studies [42].

### 4.5. Data Analysis

We tested the ability of our venom fingerprinting method to capture inter- and intra-specific variation in venom composition and generate datasets that can be used for analyses such as assessment of similarities of venom composition and correlations with ecological or genetic data. Prior to the analysis, the datasets were trimmed to contain only polymorphic markers, i.e., we excluded markers with frequency equal to 1 as they are not informative. Final binary matrices were used to calculate peak and phenotype frequencies, and other diversity measures such as individual venom richness, i.e., total number of peaks, and the Shannon diversity index [43]. To analyze patterns of venom composition we calculated pairwise Bray-Curtis similarity distances [44] among venom profiles and conducted multivariate analyses by means of non-metric multidimensional scaling (NMDS) [45]. Analyses were performed using the package *vegan* [46] in R version 3.2.3 (R Foundation for Statistical Computing, Vienna, Austria) [47] and GenAlEx 6.5b3 [48].

### 4.6. Mass Spectrometric Characterization of Venom Proteins

Molecular masses of RP-HPLC purified proteins of two representative *C. scutulatus* venoms were estimated by SDS-PAGE on 15% polyacrylamide gels under reducing conditions and stained with Coomassie Brilliant Blue G-250 (Sigma-Aldrich, St. Louis, MO, USA). Additionally, the molecular masses of the components of selected chromatographic fractions were determined by electrospray ionization (ESI) mass spectrometry using an Applied Biosystems QTrap™ 2000 mass spectrometer (Framingham, MA, USA) operated in Enhanced Multiple Charge mode in the range m/z 350–1700. Data were acquired and processed using Analyst 1.5.1. software (Framingham, MA, USA).

Protein bands of interest were excised from the gel and subjected to automated in-gel digestion using a ProGest Protein Digestion Workstation (Genomics Solutions Ltd., Cambridgeshire, UK) and sequencing-grade porcine trypsin (Promega, Madison, WI, USA). Tryptic digests were dried in a SpeedVac (Savant™, Thermo Scientific Inc., West Palm Beach, FL, USA), re-dissolved in 15 μL of 5% acetonitrile containing 0.1% formic acid, and submitted to LC-MS/MS. To this end, tryptic peptides were separated by nano-Acquity UltraPerformance LC^®^ (UPLC^®^, Waters Corporation, Milford, MA, USA) using BEH130 C18 (100 μm × 100 mm, 1.7 μm) column in-line with a Waters SYNAPT G2 High Definition Mass Spectrometry System (Waters Corporation, Milford, MA, USA). The flow rate was set to 0.6 μL/min and column was develop with a linear gradient of 0.1% formic acid in water (solution A) and 0.1% formic acid in acetonitrile (solution B), isocratically 1% B for 1 min, followed by 1–12% B for 1 min, 12–40% B for 15 min, 40–85% B for 2 min. Doubly- and triply-charged ions were selected for collision-induced dissociation (CID) MS/MS. Fragmentation spectra were interpreted manually or using the on-line form of the MASCOT program [49] against the NCBI non-redundant database [50]. MS/MS mass tolerance was set to ± 0.6 Da. Carbamidomethyl cysteine was set as fixed modification whereas propionamide cysteine and oxidation of methionine were set as variable modifications.

### 4.7. RP-HPLC Protein Fractions on the Bioanalyzer

We tested the possibility of rapidly detecting the presence of specific toxin proteins using the Bioanalyzer as an alternative to SDS-PAGE gels. The same RP-HPLC fractions used for proteomic characterization were re-suspended in ddH_2_O and run on the Bioanalyzer under reduced conditions using the Agilent Protein 80 kit. Individual bands were sized using the internal standard and the estimated molecular weights were compared with those estimated by SDS-PAGE and ESI-MS or calculated from the amino acid sequences of the best protein hits against the NCBI database.

## Figures and Tables

**Table 1:** Comparison between the Bioanalyzer on-chip technology and other available methodologies to assess variation in venom composition.

Features	On-chip	RP-HPLC	SDS-PAGE
Starting material	8–20 μg	0.5–1 mg	20–30 μg
No. of samples run simultaneously	10	1	10–30
No. of peaks/bands per venom profile (min-max)	8–17	15–36	10–20
Total preparation time	50 min	90–120 min	up to 2 days
Internal standard	yes	generally not implemented	no
Volumes of reagents	μL	L	μL-mL
Hazardous reagents	no	yes	yes
Costs of reagents per sample	3.50 € (incl. chip) 1.70 € (excl. chip)	4.14 € (incl. H_2_O) 0.94 € (excl. H_2_O)	1.50 € (precast gel) 0.43 € (homemade gel)

**Table 2:** Assignment of the reverse-phase HPLC fractions of *Crotalus scutulatus* venoms (Figure 6) to toxin families by CID-MS/MS sequencing and relative molecular masses determined by SDS-PAGE, on-chip electrophoresis with the Bioanalyzer, mass spectrometry (MS) and calculated from the amino acid sequences of the NCBI Genbank protein hits.

HPLC Fraction	Venom Type	Toxin Family	SDS-PAGE (kDa)	Bioanalyzer (kDa)	MS (Da)	aa-Derived (Da)	GenBank No.
1	A	CRISP	26	29.85	24,808.4	24,861	ACE73566
	B	CRISP	27.5	30.05		24,824	JAA97963
		PLA_2_	17.5	16.45	14,198.5	14,213	AAQ13337
		PLA_2_	13.5	11.75	13,699.4	13,647	AAM80563
2	A			No bands			
	B	CRISP	27	29.55	24,808.4	24,824	JAA97963
		PLA_2_	17	16.5	14,198.5	14,213	AAQ13337
3	A			No bands			
	B	SVSP	36	37.5		27,044	JAA98036
		PLA_2_	17	18.4	13,632.2	13,647	AAM80563
4	A	SVSP	66	109		26,701	AFJ49249
		SVSP	36	36.8		26,107	JAS04407
		PLA_2_	15	14		13,588	ANN23927
		CTL		12.9		14,864	AEU60006
	B	SVSP		36.8		26,701	AFJ49249
5	A	SVSP	36	36.8		26,107	JAS04407
		SVSP		35.7		26,599	AAL77226
		SVSP		28.3		26,444	JAA94859
		PLA_2_	15	14.4		13,588	ANN23927
		CTL		13.1		14,864	AEU60006
	B	SVSP	36	36.1		27,044	JAA98036
		SVSP	31	34.8		25,997	AEJ31998
		CTL	15	15.5		15,028	AEJ31972
		CTL	14	13.4		14,864	AEU60006
		CTL	13	11.5		14,028	AEU60003
6	A			No bands			
	B	PII-SVMP	~55	46.4		51,369	JAS04329
		PII-SVMP	28	23		51,230	JAS05301
7	A			No bands			
	B	PIII-SVMP	90	71		66,057	JAG46111
		PII-SVMP	56	57		52,457	JAG46119
		PII-SVMP	28	23.4		51,230	JAS05301
		CTL	14	14.8		14,821	AFJ49155
		CTL		13.1		14,505	JAS05338
8	A			No bands			
	B	PIII-SVMP	~116	125.4		66,001	ACV83933
		PIII-SVMP		115.5		66,001	ACV83933
		PIII-SVMP	66	66.5		66,001	ACV83933
		PIII-SVMP		63		66,001	ACV83933
		PII-SVMP	50	43.1		51,369	JAS04329
		PII-SVMP		37.7		51,309	JAS05298
		PII-SVMP	28	23.4		51,309	JAS05298
		CTL	14	14.8		14,821	AFJ49155
		CTL		13.1		14,505	JAS05338
